# National Trends in the Incidence of Hospitalization for Stroke in Japan

**DOI:** 10.31662/jmaj.2024-0311

**Published:** 2025-06-13

**Authors:** Takao Ono, Yuki Miyamoto, Tasuku Matsuyama, Chikamasa Ichita, Kasumi Satoh, Taketo Watase, Tadahiro Goto

**Affiliations:** 1 Department of Emergency Medicine and General Internal Medicine, Fujita Health University School of Medicine, Toyoake, Japan; 2 Department of Emergency Medicine, Kyoto Prefectural University of Medicine, Kyoto, Japan; 3 Department of Clinical Epidemiology and Health Economics, School of Public Health, University of Tokyo, Tokyo, Japan; 4 Gastroenterology Medicine Center, Shonan Kamakura General Hospital, Kamakura, Japan; 5 Department of Health Data Science, Yokohama City University, Yokohama, Japan; 6 Department of Emergency and Critical Care Medicine, Akita University Graduate School of Medicine, Akita, Japan; 7 TXP Medical Co., Ltd., Tokyo, Japan

**Keywords:** stroke, subarachnoid hemorrhage, intracranial hemorrhage, ischemic stroke, incidence

## Introduction

Stroke remains a leading cause of death and disability globally ^(1)^. Projections suggest that if current trends continue, stroke-related disability-adjusted life years will reach approximately 300 million by 2050 ^(1)^. Aging, in particular, is a key factor influencing stroke incidence in high-income countries ^(2)^.

In the context of global aging ^(3)^, understanding stroke incidence trends in Japan, the country with the oldest population, can offer valuable epidemiological insights. However, the actual national temporal trend in stroke hospitalization incidence remains unclear. Although several large stroke registries exist in Japan ^(4)^, they are hospital-based and may not fully represent national trends due to selection bias from hospital characteristics ^(5)^, even when using a nationwide dataset.

Nationwide, population-based administrative claims databases, such as the National Database (NDB) of Health Insurance Claims and Specific Health Checkups, include data from all hospitals in Japan, offering a more comprehensive dataset that allows us to estimate accurate stroke hospitalization trends. Therefore, we aimed to describe the national trends in stroke hospitalization incidence in Japan using NDB sampling data.

## Methods

### Study design and data source

This is a descriptive study based on NDB sampling data, publicly available from the Initiative for Clinical Epidemiological Research, covering the period from 2012 to 2019 on a quarterly basis ^(6)^. This data was randomly extracted from the NDB at a rate of 10% for inpatient records, with billing codes and stratification by age ^(6)^. The NDB is the national claims database administered by the Ministry of Health, Labour, and Welfare. Japan’s universal health insurance system allows the NDB to capture over 98% of health insurance claims. The requirements for informed consent and ethical approval were waived because the database did not contain personally identifiable information.

### Participants

We identified stroke patients using the International Classification of Diseases, Tenth Revision (ICD-10) codes from the “first admission due to this disease” field, including “subarachnoid hemorrhage (SAH),” “intracranial hemorrhage (ICH),” “ischemic stroke (IS),” and “stroke, not specified as hemorrhage or infarction.” Cases classified as “stroke, not specified as hemorrhage or infarction” were included in the IS category, as per a previous study ^(7)^. Additionally, to understand trends in stroke treatment, we identified “endovascular thrombectomy (EVT)” treatments as the use of percutaneous thrombectomy (surgical code: K178-4) ^(8)^. The correspondence table between billing codes and ICD-10 codes is provided in Table S1. We excluded the total stroke and SAH data from 2012 due to the lack of comparability in incidence with changes in the SAH billing code that occurred in 2012-2013 ^(9)^. Likewise, EVT data from 2012-2013 were excluded because insurance reimbursement for thrombectomy was established in 2014, making them non-comparable.

### Statistical analysis

First, to estimate the total number of stroke hospitalizations in the entire population, the figure was multiplied by 10, as the database consisted of a 10% random sample. Second, since the data were reported quarterly, the figure was further multiplied by 3 ^(10)^. Subsequently, we examined the number of hospitalizations for total stroke, each stroke subtype (SAH, ICH, IS), and EVT across all ages and within four age categories: 0-39 years, 40-64 years, 65-74 years, and ≥75 years (further divided into 75-84 years, 85-94 years, and ≥95 years as subcategories). We then calculated the hospitalization incidence rate per 100,000 people by age group for total stroke and each stroke subtype, across all ages and within the same age categories. We also calculated the age-adjusted incidence rate of hospitalization per 100,000 people for total stroke and each stroke subtype, using the direct method and the 2015 Japanese population as a standard ^(11)^. Additionally, we described the population and mean age for all age categories based on national census data for reference ^(11)^. All analyses were performed using R software version 4.2.0.

## Results

During the study period, the national estimate of the total number of stroke hospitalizations increased from 437,970 in 2013 to 514,920 in 2019, with similar increases observed for each stroke subtype and EVT (Table S2 and S3). The number of hospitalizations for SAH and EVT increased across all age categories, whereas hospitalizations for total stroke, ICH, and IS increased mainly in the ≥75 years category, particularly in the 75-84 and 85-94 years subcategories (Figure S1-S3, Table S3 and S4).

**Figure 1.**
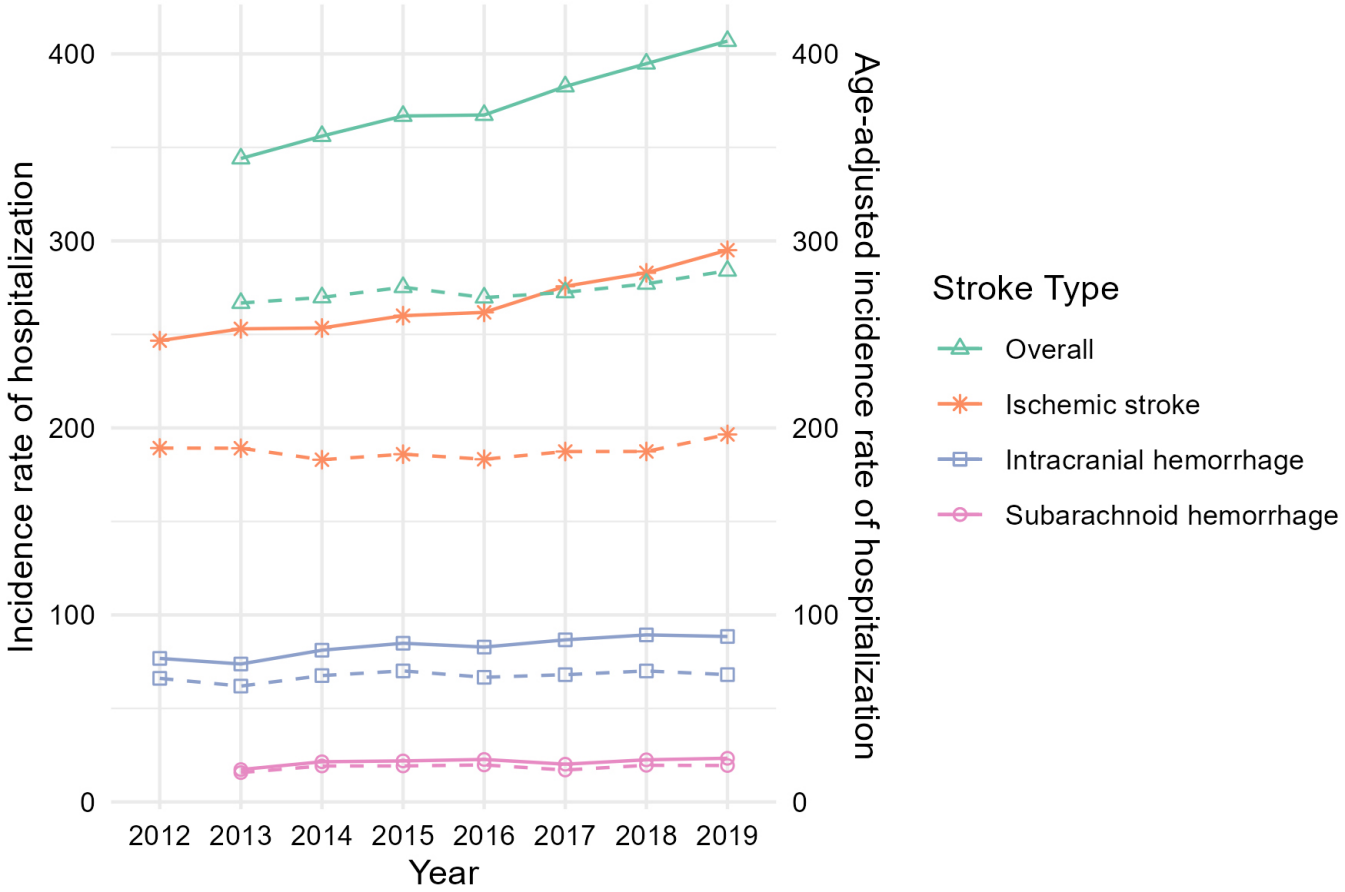
Trends in annual incidence rates of hospitalization per 100,000 people and age-adjusted incidence rates of hospitalization for each stroke subtype. Total stroke and SAH data in 2012 were excluded due to a change in the billing code. The solid line shows the incidence rate of hospitalization per 100,000, and the dashed line shows the age-adjusted incidence rate of hospitalization. SAH: subarachnoid hemorrhage.

**Figure 2.**
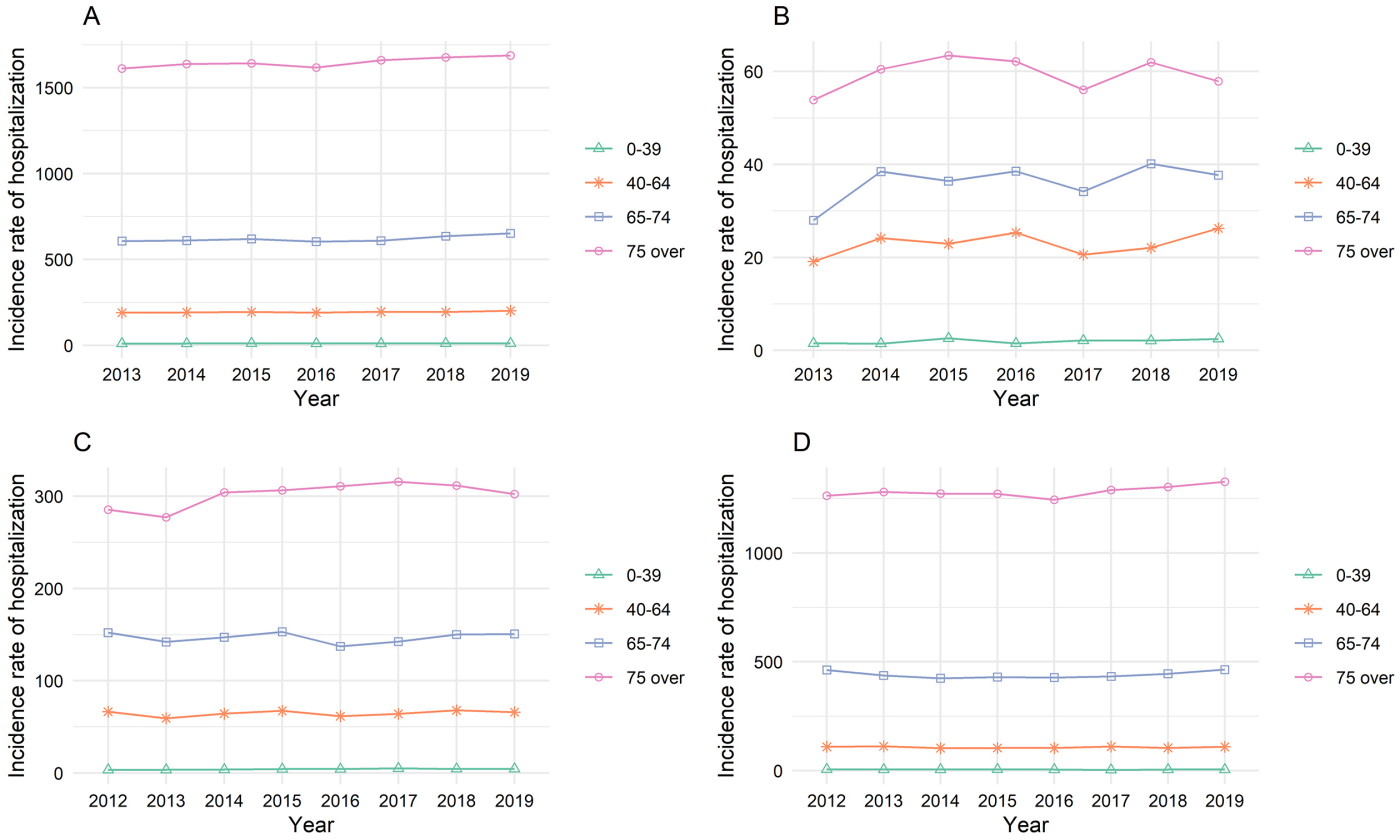
Trends in annual incidence rates of hospitalization per 100,000 people respective of age groups for each stroke subtype by age groups. A. Total stroke; B. Subarachnoid hemorrhage; C. Intracranial hemorrhage; D. Ischemic stroke Total stroke and SAH data in 2012 were excluded due to changes in the billing code. SAH: subarachnoid hemorrhage.

The hospitalization incidence rate per 100,000 people increased from 344.1 in 2013 to 406.9 in 2019 for total stroke, from 17.3 to 23.3 for SAH, from 76.7 to 88.5 for ICH, and from 246.7 to 295.1 for IS (Figure 1 and Table S5). Within the distinct age categories, the hospitalization incidence rate in 2018 was higher than in 2013, mainly for patients aged ≥75 years (Figure 2 and Table S6).

The age-adjusted hospitalization incidence rate per 100,000 increased slightly from 266.8 to 284.1 for total stroke and increased for each stroke subtype (Figure 1 and Table S7).

The census data showed little change in the total population during the study period; however, the proportion of individuals aged 65-74 and ≥75 increased from 12.2% to 13.8% and from 11.9% to 14.7%, respectively. The average age of the population in the ≥75 age category consistently increased (Table S8).

## Discussion

Using NDB sampling data, we demonstrated an overall increase in both the number and incidence of stroke hospitalizations in Japan, contrary to previous studies reporting a decline in stroke incidence ^(12)^. The increasing trend was particularly evident in the ≥75 age category. Considering the census data, this rise may be attributed to the aging population, specifically the growth in the older population and the higher mean age within each age group.

Although stroke prevention research has advanced, effective prevention in older adults remains challenging, such as ensuring appropriate anticoagulation therapy and more individualized, intensive blood pressure reduction ^(13)^. Despite progress in reducing mortality ^(1)^, the increasing number of stroke hospitalizations could be linked to the rising number of patients surviving with long-term disabilities, leading to greater demand for long-term care, higher costs, and a greater burden on families ^(14)^. In Japan, implementing strategies tailored to an aging population is crucial.

This study had several limitations. First, it relied on claims data, which may be prone to misclassification. However, the positive predictive value of the ICD-10 code for stroke is high (86.4%) ^(15)^, and the impact is likely consistent over time. Second, the database lacked individual data, limiting detailed analysis of mortality, functional outcomes, and severity. Although stroke functional outcomes at hospital discharge have shown little change over time ^(4)^, we cannot rule out the possibility of an increase in minor stroke hospitalizations due to changes in diagnostic technology or admission criteria. To address the uncertainties, further detailed analyses are needed, such as combining existing databases.

In conclusion, this study showed that both the number of hospitalizations and the incidence rate of stroke hospitalization increased in Japan from 2013 to 2019. The age-adjusted incidence rate also rose during this period. These findings highlight a concerning trend in stroke occurrence and management in the aging population, emphasizing the need for enhanced preventive strategies and healthcare planning.

## Article Information

### Conflicts of Interest

None

### Acknowledgement

We thank Yasuyuki Okumura of the Initiative for Clinical Epidemiological Research for establishing the NDB sampling data.

### Author Contributions

Takao Ono is responsible for the paper. Takao Ono, Yuki Miyamoto, Chikamasa Ichita, and Tadahiro Goto conceived the study. Taketo Watase and Tadahiro Goto supervised, and Yuki Miyamoto, Tasuku Matsuyama, Chikamasa Ichita, Kasumi Satoh, and Tadahiro Goto provided statistical advice. Takao Ono drafted the manuscript. All authors revised it substantially.

### Approval by Institutional Review Board (IRB)

None.

### Data Availability

The raw data supporting the findings of this study can be accessed through the following link: https://icer.tokyo/materials/ndb_feasibility_check/.

## Supplement

Supplemental Materials
