# Supplementary material for: National Trends in the Incidence of Hospitalization for Stroke in Japan
Source: JMA J. 2025 Jun 13;8(3):970–3. doi: 10.31662/jmaj.2024-0311 (PMC12328453; doi:10.31662/jmaj.2024-0311)
Supplement: Supplemental Materials [file 2433-3298-8-3-0970-s001.pdf]

SUPPLEMENTAL MATERIALS

Figure S1. Trends in the number of hospitalizations for each stroke subtype by age groups.

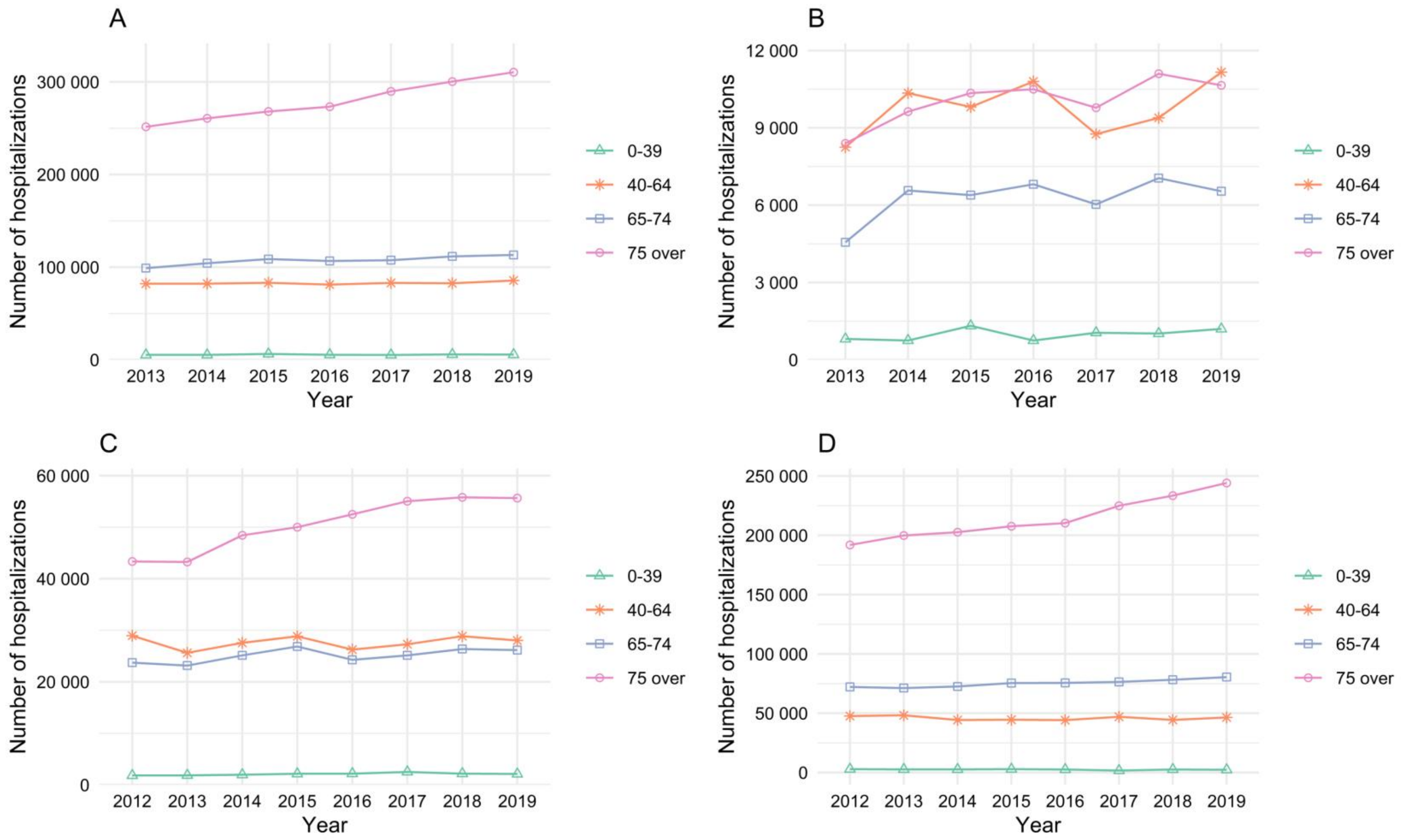

A. Total stroke; B. Subarachnoid hemorrhage; C. Intracranial hemorrhage; D. Ischemic stroke

Total stroke and Subarachnoid hemorrhage data in 2012 were excluded due to changes in the billing code.

Figure S2. Trends in the number of hospitalizations for each stroke subtype by detailed age groups.

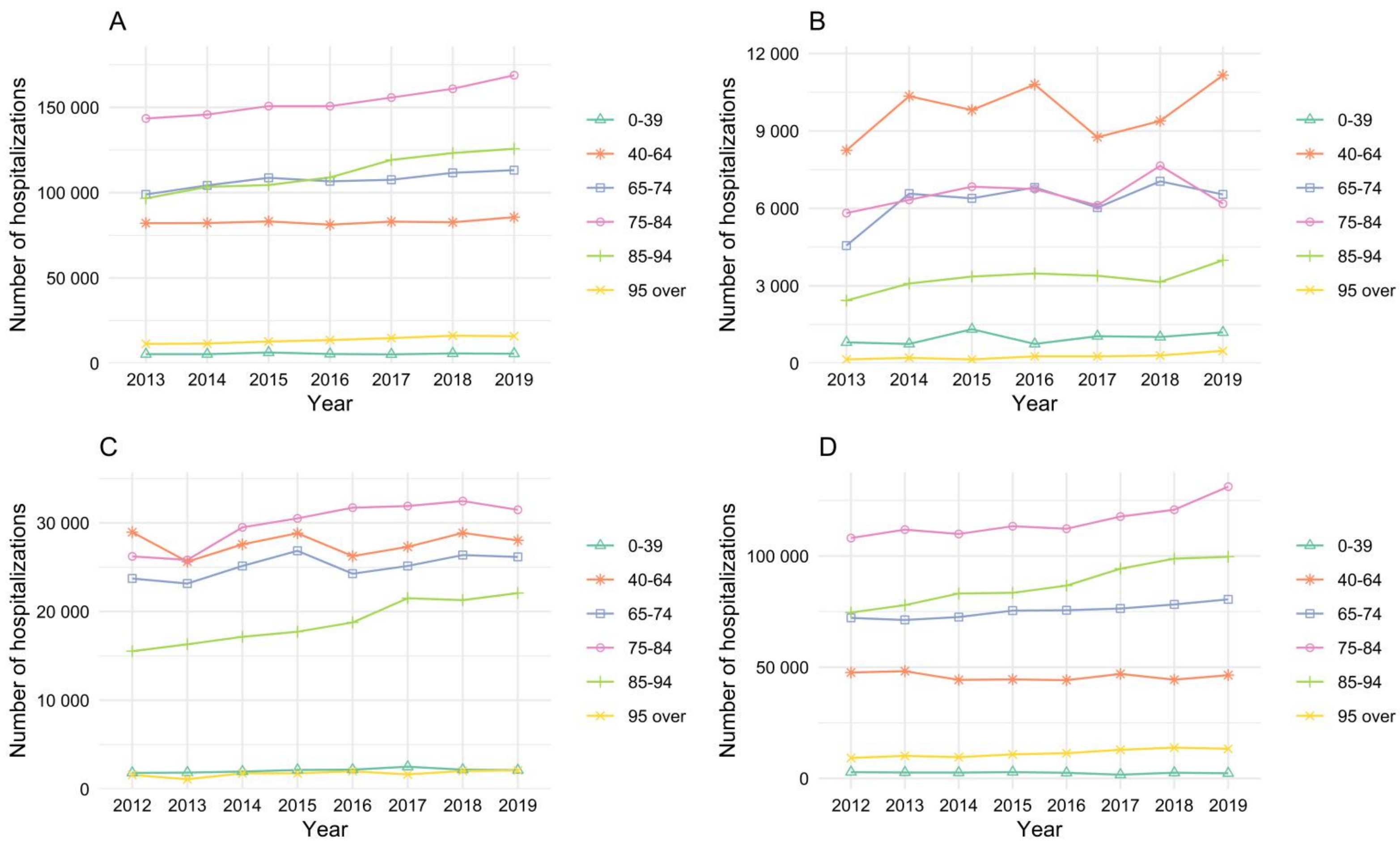

A. Total stroke; B. Subarachnoid hemorrhage; C. Intracranial hemorrhage; D. Ischemic stroke

Total stroke and Subarachnoid hemorrhage data in 2012 were excluded due to changes in the billing code.

Figure S3. Trends in the number of endovascular thrombectomy.

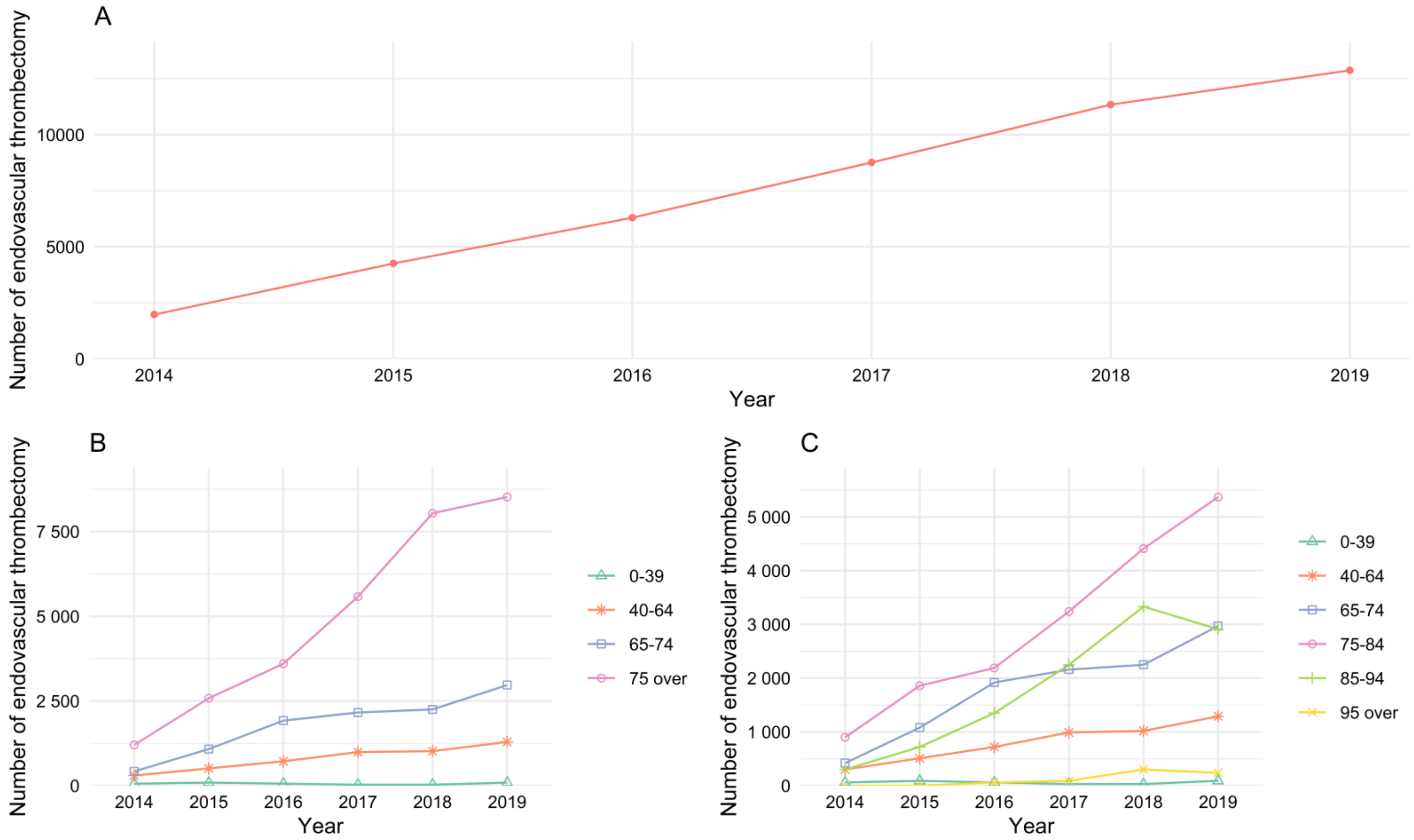

A. Total; B. By age groups; C. By detailed age groups

Table S1. Billing codes used for identifying diagnoses of each stroke subtype, along with their corresponding ICD-10 codes.

Types of stroke	ICD-10	Billing code
Subarachnoid hemorrhage	I600	8847449, 8847541, 8849043, 8851036
	I601	8847527
	I602	8847505
	I603	8847468
	I604	8847545
	I605	8847531, 8849042
	I606	8847469, 8847506, 8851076, 8851101, 8851120
	I607	8847536
	I608	8835797, 8847895, 8847896
	I609	4309001, 4309005, 8836339, 8838751
Intracranial hemorrhages	I610	4310038, 4319027, 4319030, 8847680
	I611	8839257
	I613	4319013, 8841358, 8845147
	I614	4319006
	I615	4319018, 4319032
	I616	8836998
	I618	4320007
	I619	4319003, 4319009, 4319020, 8847897, 8850906
Ischemic stroke	I630	8838690, 8846410, 8851143, 8851154, 8851160
	I631	8838691, 8851144, 8851155, 8851161
	I632	8838692, 8851142, 8851145, 8851153, 8851156, 8851159, 8851162
	I633	8842255, 8846351, 8846384, 8846385, 8851061, 8851062, 8851063, 8851073, 8851079, 8851098, 8851117, 8851123, 8851138

	I634	8842272, 8846397, 8846412, 8846413, 8846415, 8846416, 8847851, 8850530, 8851074, 8851080, 8851099, 8851118, 8851124, 8851126, 8851127, 8851128, 8851139
	I635	4330012, 4341002, 4341005, 4341010, 4341027, 4341052, 4341053, 8838703, 8846357, 8846373,8846419, 8846436, 8851072, 8851075, 8851078, 8851081, 8851097, 8851100, 8851116, 8851119, 8851122, 8851125, 8851137,8851140
	I636	3259015, 8835486
	I638	4341018, 4341044, 4341045, 4341049, 4341056, 8846420, 8846438, 8846450, 8846451, 8848096
	I639	4349005, 4369016, 8838708, 8838753, 8846439, 8849423, 8849460
Stroke, not specified as hemorrhage or infarction	I64	4369009, 4369014, 8838704

Table S2. Trends in annual number of hospitalizations for each stroke subtype.

	Year							
	2012	2013	2014	2015	2016	2017	2018	2019
Total		437,970	452,490	466,200	466,650	485,580	500,400	514,920
Subarachnoid hemorrhage		22,020	27,300	27,870	28,860	25,620	28,560	29,550
Intracranial hemorrhage	97,860	93,900	103,110	107,850	105,210	110,010	113,220	111,960
Ischemic stroke	314,520	322,050	322,080	330,480	332,580	349,950	358,620	373,410

Table S3. Trends in annual number of endovascular thrombectomy.

		Year					
	Age group	2014	2015	2016	2017	2018	2019
Total		1,980	4,260	6,300	8,760	11,340	12,870
Endovascular thrombectomy	0–39 years	60	90	60	30	30	90
	40–64 years	300	510	720	990	1,020	1,290
	65–74 years	420	1,080	1,920	2,160	2,250	2,970
	≥75 years	1,200	2,580	3,600	5,580	8,040	8,520
	75–84 years	900	1,860	2,190	3,240	4,410	5,370
	85–94 years	300	720	1,350	2,250	3,330	2,910
	≥95 years	0	0	60	90	300	240

Table S4. Trends in annual number of hospitalizations for each stroke subtype by age groups.

		Year							
Stroke type	Age group	2012	2013	2014	2015	2016	2017	2018	2019
Total	0–39 years		5,340	5,340	6,330	5,460	5,250	5,790	5,640
	40–64 years		82,110	82,230	83,160	81,240	83,040	82,620	85,620
	65–74 years		98,970	104,250	108,690	106,680	107,550	111,630	113,190
	≥75 years		251,550	260,670	268,020	273,270	289,740	300,360	310,470
	75–84 years		143,490	145,740	150,750	150,720	155,760	160,920	168,840
	85–94 years		96,660	103,380	104,520	108,930	119,190	123,270	125,730
	≥95 years		11,400	11,550	12,750	13,620	14,790	16,170	15,900
Subarachnoid hemorrhage	0–39 years		810	750	1,320	750	1,050	1,020	1,200
	40–64 years		8,250	10,350	9,810	10,800	8,760	9,390	11,160
	65–74 years		4,560	6,570	6,390	6,810	6,030	7,050	6,540
	≥75 years		8,400	9,630	10,350	10,500	9,780	11,100	10,650
	75–84 years		5,820	6,330	6,840	6,750	6,120	7,650	6,180
	85–94 years		2,430	3,090	3,360	3,480	3,390	3,150	3,990
	≥95 years		150	210	150	270	270	300	480
Intracranial hemorrhage	0–39 years	1,830	1,860	1,980	2,160	2,190	2,520	2,190	2,130
	40–64 years	28,950	25,620	27,570	28,830	26,250	27,300	28,860	28,020
	65–74 years	23,730	23,160	25,140	26,850	24,270	25,140	26,370	26,160
	≥75 years	43,350	43,260	48,420	50,010	52,500	55,050	55,800	55,650
	75–84 years	26,220	25,830	29,490	30,510	31,710	31,890	32,460	31,470
	85–94 years	15,540	16,320	17,160	17,730	18,780	21,510	21,300	22,080
	≥95 years	1,590	1,110	1,770	1,770	2,010	1,650	2,040	2,100
Ischemic stroke	0–39 years	2,850	2,670	2,610	2,850	2,520	1,680	2,580	2,310
	40–64 years	47,610	48,240	44,310	44,520	44,190	46,980	44,370	46,440
	65–74 years	72,180	71,250	72,540	75,450	75,600	76,380	78,210	80,490

≥75 years	191,880	199,890	202,620	207,660	210,270	224,910	233,460	244,170
75–84 years	108,060	111,840	109,920	113,400	112,260	117,750	120,810	131,190
85–94 years	74,610	77,910	83,130	83,430	86,670	94,290	98,820	99,660
≥95 years	9,210	10,140	9,570	10,830	11,340	12,870	13,830	13,320

Table S5. Trends in stroke incidence rates of hospitalization per 100,000 people respective of age groups for each stroke subtype.

	Year							
	2012	2013	2014	2015	2016	2017	2018	2019
Total		344.1	356.1	366.8	367.3	382.6	394.8	406.9
Subarachnoid hemorrhage		17.3	21.5	21.9	22.7	20.2	22.5	23.3
Intracranial hemorrhage	76.7	73.8	81.1	84.9	82.8	86.7	89.3	88.5
Ischemic stroke	246.7	253.0	253.4	260.0	261.8	275.7	282.9	295.1

Table S6. Trends in annual incidence rates of hospitalization per 100,000 people respective of age groups for each stroke subtype by age groups.

		Year							
Stroke type	Age group	2012	2013	2014	2015	2016	2017	2018	2019
Total	0–39 years		10.2	10.4	12.6	11.0	10.7	11.9	11.7
	40–64 years		190.1	192.0	194.3	190.5	195.2	194.4	201.4
	65–74 years		607.4	610.3	619.5	603.8	609.6	635.5	652.3
	≥75 years		1612.1	1637.6	1642.0	1617.8	1660.9	1676.8	1687.2
Subarachnoid hemorrhage	0–39 years		1.6	1.5	2.6	1.5	2.1	2.1	2.5
	40–64 years		19.1	24.2	22.9	25.3	20.6	22.1	26.2
	65–74 years		28.0	38.5	36.4	38.5	34.2	40.1	37.7
	≥75 years		53.8	60.5	63.4	62.2	56.1	62.0	57.9
Intracranial hemorrhage	0–39 years	3.4	3.6	3.9	4.3	4.4	5.1	4.5	4.4
	40–64 years	66.5	59.3	64.4	67.4	61.6	64.2	67.9	65.9
	65–74 years	152.1	142.1	147.2	153.0	137.4	142.5	150.1	150.8
	≥75 years	285.3	277.2	304.2	306.4	310.8	315.6	311.5	302.4
Ischemic stroke	0–39 years	5.4	5.1	5.1	5.7	5.1	3.4	5.3	4.8
	40–64 years	109.3	111.7	103.5	104.0	103.6	110.4	104.4	109.2
	65–74 years	462.7	437.3	424.7	430.0	427.9	432.9	445.2	463.8
	≥75 years	1263.0	1281.0	1272.9	1272.2	1244.9	1289.3	1303.3	1326.9

Table S7. Age-adjusted incidence rate of hospitalization for each stroke subtype.

	Year							
Stroke type	2012	2013	2014	2015	2016	2017	2018	2019
Total		266.8	269.8	275.3	269.7	272.5	277.0	284.1
Subarachnoid hemorrhage		15.7	19.2	19.3	19.8	17.1	19.6	19.5
Intracranial hemorrhages	65.5	61.9	67.6	70.1	66.7	68.0	70.0	68.1
Ischemic stroke	186.3	189.1	183.0	185.9	183.3	187.4	187.4	196.5

Table S8. Trends in population estimates per 100,000 and mean age by age groups in Japan.

Age group		2012	2013	2014	2015	2016	2017	2018	2019
Total	Number	127,515	127,298	127,083	127,095	126,933	126,706	126,443	126,167
0–39 years	Number (%)	53,170 (41.7)	52,194 (41.0)	51,255 (40.3)	50,438 (39.7)	49,737 (39.2)	49,101 (38.8)	48,505 (38.4)	47,959 (38.0)
	Mean age	21.7	21.5	21.4	21.4	21.3	21.2	21.2	21.2
40–64 years	Number (%)	43,550 (34.2)	43,205 (33.9)	42,824 (33.7)	42,790 (33.7)	42,604 (33.6)	42,454 (33.5)	42,362 (33.5)	42,321 (33.5)
	Mean age	52.2	51.9	51.7	51.6	51.5	51.4	51.4	51.5
65–74 years	Number (%)	15,601 (12.2)	16,298 (12.8)	17,083 (13.4)	17,545 (13.8)	17,683 (13.9)	17,670 (14.0)	17,603 (13.9)	17,394 (13.8)
	Mean age	69.3	69.2	69.1	69.2	69.2	69.2	69.3	69.4
≥75 years	Number (%)	15,191 (11.9)	15,601 (12.3)	15,916 (12.5)	16,321 (12.8)	16,907 (13.3)	17,481 (13.8)	17,977 (14.2)	18,490 (14.7)
	Mean age	81.8	81.9	82.1	82.1	82.1	82.2	82.2	82.3
75–84 years	Number (%)	10,884 (8.5)	11,064 (8.7)	11,137 (8.8)	11,380 (9.0)	11,705 (9.2)	12,031 (9.5)	12,281 (9.7)	12,570 (10.0)
85–94 years	Number (%)	3,926 (3.1)	4,140 (3.3)	4,368 (3.4)	4,518 (3.6)	4,753 (3.7)	4,978 (3.9)	5,188 (4.1)	5,372 (4.3)
≥95 years	Number (%)	381 (0.3)	397 (0.3)	411 (0.3)	423 (0.3)	449 (0.4)	472 (0.4)	508 (0.4)	548 (0.4)
